# Paraproteinaemic keratopathy simulating a crystalline keratopathy

**DOI:** 10.1186/s12886-024-03487-6

**Published:** 2024-06-19

**Authors:** Andrea Aramburu-González, Silvia López-Plandolit Antolin, Jose Antonio Márquez-Navarro

**Affiliations:** 1 Department of Ophthalmology, Basurto University Hospital, Avda. Montevideo s/n, Bilbao, E- 48013 Spain; 2 Department of Haematology, Basurto University Hospital, Bilbao, Spain

**Keywords:** Crystalline keratopathy, Paraproteinemia, Monoclonal gammopathy of undetermined significance, Corneal deposit, Anterior segment optical coherence tomography

## Abstract

**Background:**

Paraproteinemic keratopathy is a rare disorder characterized by the bilateral accumulation of polychromatic deposits diffusely in all corneal layers together or not with diffuse or patchy pseudo lipid deposits. We present an atypical case of paraproteinemic keratopathy which lead to an initial misdiagnosis of infectious crystalline keratopathy.

**Case presentation:**

a 69-year-old woman with an asymptomatic keratopathy detected during a cataract intervention. Slit-lamp examination revealed several hyper refringent subepithelial foci with fern-shaped branches, resembling crystalline keratopathy, in her left eye. Anterior segment optical coherence tomography revealed exclusively subepithelial hyperreflective lesions limited to the anterior stroma. The progressive bilateralization and progression of the condition prompted us to include other entities with crystalline corneal deposits in our differential diagnosis. Hematological analysis showed a high number of free Kappa light chains. Despite the typical clinical appearance of crystalline keratopathy, the atypical evolution and test results led us to consider that monoclonal gammopathy could be the cause of this entity.

**Conclusions:**

Paraproteinemic keratopathy may present in its early stages as a unilateral subepithelial crystalline keratopathy. Thus, it must always be taken into account in the differential diagnosis of any crystalline keratopathy, particularly when there are no predisposing factors for an infectious crystalline keratopathy. Early recognition of this rare entity is important to address the associated potentially serious systemic disease.

## Background

Crystalline keratopathy is a rare but serious clinical entity that can be secondary to a wide variety of causes, from topical medications to systemic diseases [1, 2]. Once the diagnosis of this entity is established, it is important to carry out an adequate investigation to determine its aetiology and indicate the most appropriate treatment [1]. The most frequent cause of crystalline keratopathy is infectious aetiology, the rarest being lymphoproliferative disorders.

Paraproteinemia, also known as monoclonal gammopathy, is due to the presence of a monoclonal protein in the blood. Monoclonal gammopathy of undetermined significance (MGUS) is the most common form of paraproteinemia. Paraproteins can be deposited in the cornea, which is known as paraproteinemic keratopathy (PPK), also called corneal crystalline deposition, MGUS keratopathy, or MGUS associated corneal opacification [1]. Corneal crystalline deposits may be the first clinical symptom of monoclonal gammopathy, resulting in the loss of visual acuity.

Infectious crystalline keratopathy (ICK) was first reported by Gorovoy et al. in 1983, when they identified bacteria colonizing the cornea after penetrating keratoplasty [2]. ICK is a rare and characteristic, but not exclusive, manifestation of Streptococcus mitis infectious keratitis. It has been defined as an indolent infectious keratitis with characteristic branching needle-shaped opacities and absence of corneal or anterior segment inflammation [2, 3]. A dramatic increase in ICK cases was recorded with the increase in the number of penetrating keratoplasties. ICK occurs predominantly in adults, most commonly unilaterally. There does not seem to be a predominance of sex or race. Microbial colonies commonly aggregate in the anterior stroma or middle stroma [3]. When ICK is suspected, a corneal culture should be performed to identify the etiological pathogen. Superficial corneal scraping is often unsuccessful as this technique ay not go to the full depth of the lesions and a corneal biopsy may be required to confirm the diagnosis. The most frequent pathogen in ICK is S. viridans [3, 4].

Branching needle-like opacities in the absence of corneal or anterior segment inflammation are considered characteristic of ICK [4, 5]. A history of previous epithelial defect, surgical procedures or topical corticosteroids are also important considerations in the diagnosis of ICK. However, several other conditions can mimic the appearance of ICK. Therefore, it is necessary to carry out an exhaustive clinical history, with special attention to entities such as gout, cystinosis and multiple myeloma. Topical medications should also be recorded, as some can cause crystal deposition in the superficial corneal stroma. Ophthalmologic examination should exclude corneal dystrophies, lipid keratopathy, and other infectious keratitis. The use of anterior segment optical coherence tomography (AS-OCT) is very useful, because it helps to make a differential diagnosis, depending on the location of the deposits in the different layers of the cornea [6].

Treatment of ICK involves aggressive, prolonged antibiotic therapies, due to the biofilm generated by the microorganism, drugs are usually less efficient. A penetrating keratoplasty may be required in refractory cases (therapeutic keratoplasty) and in cases of scars that affect the visual axis (optical keratoplasty) [5].

## Case presentation

**Fig. 1:**
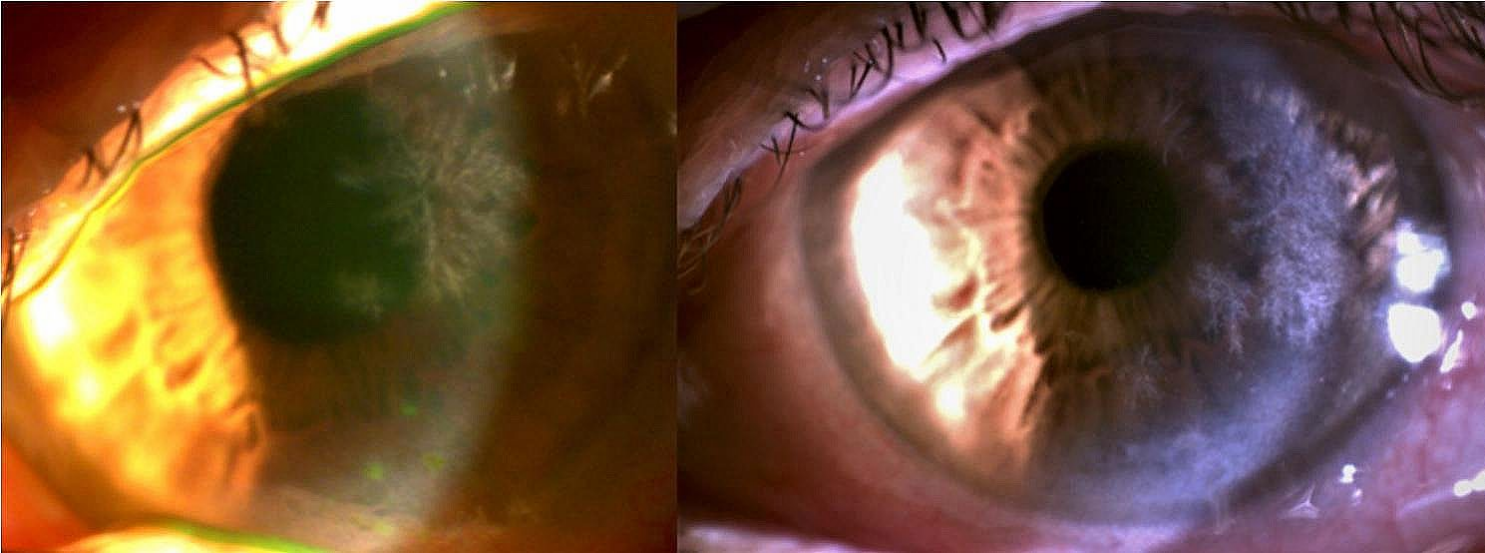
Biomicroscopy of the left eye. Infiltration of white-gray opacities within the corneal stroma, appearing as stellate or branching fernlike opacities (left) and extension of the lesions to the central and peripheral level 360º in a period of 4 months (right)

**Fig. 2:**
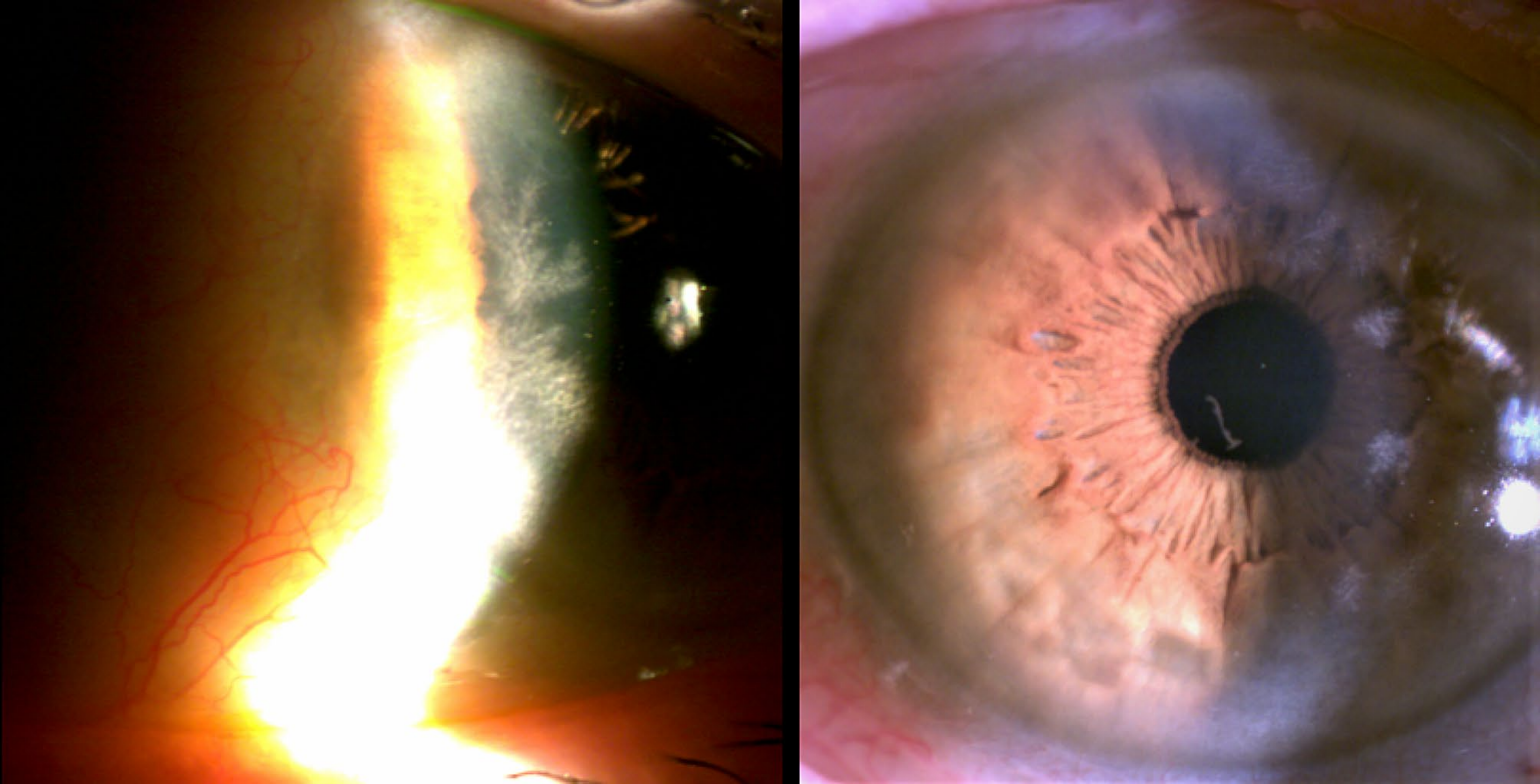
Biomicroscopy of the Right eye. Infiltration of white-gray opacities within the corneal stroma, appearing as stellate or branching fernlike opacities (left) and extension of the lesions to the central and peripheral level 360º in a period of 4 months (right)

We report a rare case of a 69-year-old woman with asymptomatic keratopathy first detected during cataract surgery on the left eye. Note that previous examinations were normal. The patient’s visual acuity after cataract surgery of the left eye was 1’0. Slit-lamp examination of the left eye revealed several hyper refringent subepithelial foci with fern-shaped branches (Fig. 1). Corneal scraping was performed, giving a negative result. Corneal samples obtained later in the operating room after performing a keratectomy and direct scraping of the lesions, were positive for *Cutinebacterium acnes*, which was sensitive to vancomycin, ciprofloxacin, and minocycline. No clinical response was observed though with topical treatment with vancomycin and ciprofloxacin. In the following 5 months, white-gray corneal opacities lesions progressed in extension in her left eye and other identical ones appeared in her right eye (Fig. 2), initially with peripheral location and later also paracentral and 360º in both eyes. The patient was still asymptomatic, presenting visual acuity of 1’0 in the cataract operated eye and 0’6 in the right eye, due to cataract.

**Fig. 3:**
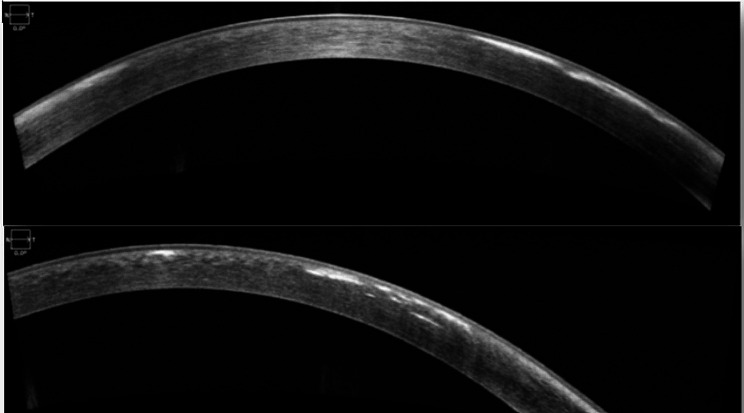
Optical coherence tomography of the anterior segment (AS-OCT) of the right eye (above) and left eye (down) shows hyper-reflective lesions exclusively subepithelial and limited to the anterior stroma

**Fig. 4:**
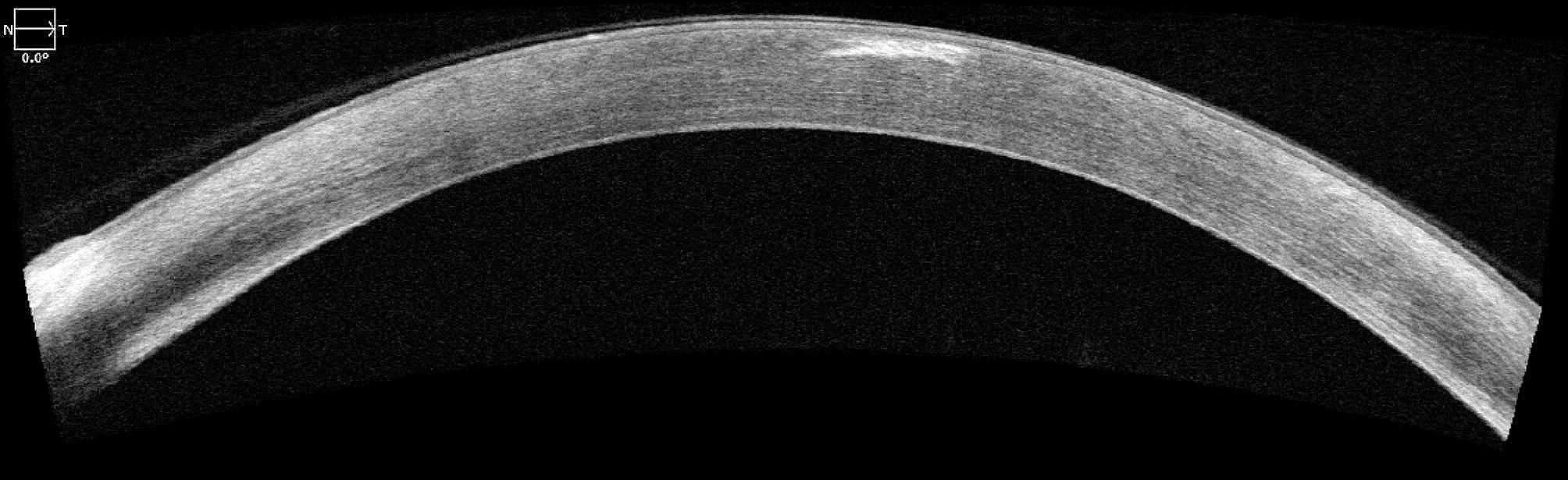
Optical coherence tomography of the anterior segment (AS-OCT) of the left eye shows central sub-epithelial hyperreflective lesions

AS-OCT (ZEISS Cirrus™ HD-OCT 5000) was performed, which revealed hyperreflective subepithelial lesions limited to the anterior stroma in the periphery (Fig. 3) and in the centre of the cornea (Fig. 4).

Although at first the appearance and location of the lesions led us to think of an infectious crystalline keratopathy, the bilaterality, the absence of any ophthalmologic history and progression of the lesions made us reconsider the initial diagnosis of ICK and look for other entities with crystalline corneal deposits such as gout and paraproteinemia. The age of the patient at the time of appearance of the lesions led us to rule out a dystrophic origin and cystinosis.

The blood test that we performed after diagnosing the corneal deposits and observing the evolution of the condition, revealed a high number of free kappa light chains. The patient was referred to Hematology, where the diagnosis of MGUS was established. Haematologists did not consider any systemic treatment necessary. The rapid progression of the corneal deposits obliged us to establish a closer ophthalmological follow-up. In this way, if corneal deposits appear in the central cornea or if visual acuity is affected, the haematologist would be notified immediately. The presence of kappa light chains in the blood analysis prompted us to consider if the etiology of the corneal lesions could be due to paraproteinemia.

## Discussion

MGUS is a proliferative plasma cell disorder, characterized by the presence of a monoclonal (M) protein spike of ≤ 30 g/l, a plasma cell content of < 10% in bone marrow, and the absence of multiple myeloma or related malignant lymphoplasmacytic neoplasms (MLN) [7].

Patients with MGUS and multiple myeloma have been reported to develop paraproteinemic crystalline keratopathy in 1% of cases. Monoclonal gammopathy is a rare cause of crystalline keratopathy, but the possibility of a lymphoproliferative disorder must be taken into account in the presence of clinical findings that do not fit the diagnosis of another crystalline keratopathy [7]. In the present case, the appearance and location of the corneal lesions led us to suspect infectious crystalline keratopathy as the main diagnosis, however, due to bilaterality, the absence of any ophthalmologic history and evolution, we had to perform a differential diagnosis with other entities such as corneal dystrophies, gout, cystinosis, drugs such as fluoroquinolones and paraproteinemia.

It was after the analytical finding of elevated Kappa light chains that we suspected that our clinical case could be a PPK.

It is unknown why patients with MGUS develop corneal deposits. It has been postulated that these crystalline deposits may be delivered from limbal vessels to the cornea. Theoretically, variation in biochemical properties of the M protein and differences in limbal vascular properties that contribute to the transport of circulating M protein to the cornea, may explain the variability in corneal deposit formation among MGUS patients [1].

Primary signs in crystalline keratopathy in patients with MGUS include corneal crystalline deposits and corneal opacity. Patients may report mild visual signs, such as photophobia, but their visual acuity is usually not affected. Severe cases of paraproteinemic crystalline keratopathy are rare but may result in corneal endothelial decompensation with progressive corneal thickening, stromal haze, and visual loss [1, 9]. In the present case, the patient did not present changes in visual acuity due to corneal lesions.

Corneal crystalline deposits may be the first clinical signs of monoclonal gammopathy. PPK is characterized by the accumulation of bilateral polychromatic deposits diffusely in all corneal layers together with diffuse or patchy pseudo lipid deposits [1, 8–10]. However, in the present case, the characteristics of the corneal deposits did not match with those usually reported for the PPK. To date only subepithelial and anterior stromal corneal involvement can be observed, and all deposits are still fern-like and not polychromatic. To our knowledge such an atypical manifestation of PPK has not been previously reported.

If bilateral corneal deposits appear in a healthy patient, a haematological study is indicated to determine circulating M protein, and, if present, to exclude a malignant plasma clone indicative of multiple myeloma [9].

MGUS is a benign form of paraproteinemia. However, 10–18% of MGUS patients can develop multiple myeloma, macroglobulinemia, amyloidosis or lymphoma over the years [10]. A systemic therapy is not indicated in MGUS, but annual haematological and blood analysis is advisable, as findings of ocular dysfunction may be an indication for initiation of systemic therapy. In symptomatic patients with paraproteinemic crystalline keratopathy, treatment of the underlying disorder is the mainstay. Systemic treatments are varied and may include plasma exchange, rituximab, chemotherapy (alkylating agents, purine analogues, bortezomib and thalidomide) and stem cell transplantation. It is important to mention that resolution of the underlying disease may slow the progression of clinical manifestations [10]. After treating systemic disease, treatment of PPK is only necessary when vision is significantly impaired. There are different therapies that can be used for the treatment of PPK, including: phototherapeutic keratectomy (PTK), penetrating keratoplasty (PK) or deep anterior lamellar keratoplasty (DALK) [8]. Primary keratoprosthesis implantation is an option as a second therapeutic line. It is also important to mention that the use of topical corticosteroids has not been shown to be effective in slowing the progression of PPK. It is important to mention that PPK may appear again in keratoplasty if paraprotein levels remain elevated [6, 8]. In the present case, the patient’s visual acuity was preserved, despite having corneal lesions that reached the visual axis, which is why we consider the present case to be moderate. However, if the patient had a decrease in visual acuity, corneal treatments such as PTK and/or DALK would have to be considered, depending on the depth of the lesions.

In the present case, the patient has had a visual acuity of 1’0 in left eye during the entire follow-up, and visual acuity of 0’6 in right eye due to cataract, being asymptomatic, so it has not been necessary to evaluate any ocular treatment for the moment. We would consider starting systemic treatment if our patient started to have clinical symptoms. If systemic treatment does not stabilize the patient, we would consider treatments such as PTK, PK and DALK depending on visual acuity.

In this present case, despite a typical biomicroscopic appearance of infectious crystalline keratopathy with positive cultures, the absence of any prior ophthalmologic history and a completely atypical evolution raised a red flag and obliged us to consider other causes of crystalline keratopathy.

## Conclusions

Although PPK is an infrequent manifestation of monoclonal gammopathy, and is not usually mistaken for infectious crystalline keratopathy, it should be considered even in cases of unilateral and subepithelial corneal involvement, particularly when there are no predisposing factors for an infectious crystalline keratopathy. Early recognition of this rare entity is important to address the associated potentially serious systemic disease.

## Data Availability

Data sharing is not applicable to this article as no datasets were generated or analysed during the current study.

## Abbreviations

MGUS: Monoclonal Gammopathy of Undetermined Significance
PPK: Paraproteinemic keratopathy
ICK: Infectious crystalline keratopathy
AS-OCT: Anterior segment optical coherence tomography
MLN: Malignant lymphoplasmacytic neoplasms
